# Immediate Hemodynamic Responses to Transcutaneous Electrical Diaphragmatic Stimulation in Critically Ill Elderly Patients

**DOI:** 10.1155/2021/9091278

**Published:** 2021-12-22

**Authors:** Hebert Olímpio Júnior, Gustavo Bittencourt Camilo, Aline Priori Fioritto, Agnaldo José Lopes

**Affiliations:** ^1^Medical Sciences Post-Graduation Program, School of Medical Sciences, State University of Rio de Janeiro (UERJ), Rio de Janeiro, Brazil; ^2^Faculty of Medical and Health Sciences of Juiz de Fora (SUPREMA), Minas Gerais, Brazil; ^3^Federal University of Juiz de Fora (UFJF), Juiz de Fora, Minas Gerais, Brazil; ^4^Rehabilitation Sciences Post-Graduation Program, Augusto Motta University Center (UNISUAM), Rio de Janeiro, Brazil; ^5^Local Development Post-Graduation Program, Augusto Motta University Center (UNISUAM), Rio de Janeiro, Brazil

## Abstract

**Background:**

Critically ill patients admitted to intensive care units (ICUs) may develop diaphragmatic dysfunction, especially when artificial airways are used. Positive effects have been observed when using the transcutaneous electrical diaphragmatic stimulation (TEDS) technique in different clinical conditions. However, no study has evaluated the safety of TEDS in patients admitted to ICUs. This study is aimed at evaluating the influence of TEDS on the hemodynamic and vital parameters of critically ill elderly patients under invasive mechanical ventilation (IMV).

**Methods:**

Forty-seven patients aged >60 years under IMV were evaluated for hemodynamic variables before and after TEDS. The procedure lasted 30 minutes and was performed once.

**Results:**

The sample consisted of 33 men and 14 women with a mean age of 69.9 ± 7.64 years. The mean systolic blood pressures pre-TEDS and post-TEDS were 126.6 ± 23.7 and 122.9 ± 25.9, respectively (*p* = 0.467). The mean diastolic blood pressures pre-TEDS and post-TEDS were 71.1 ± 12.2 and 67.7 ± 14.2, respectively (*p* = 0.223). No significant differences in the mean arterial pressure or heart rate were found between the pre-TEDS and post-TEDS time points (*p* = 0.335 and *p* = 0.846, respectively).

**Conclusion:**

Our findings suggest that TEDS does not have clinically relevant impacts on hemodynamic or vital parameters in critically ill elderly patients. These findings point to the possible safety of TEDS application in this population.

## 1. Introduction

Critically ill patients admitted to intensive care units (ICUs) are prone to develop muscle weakness, especially when they use artificial airways. The factors associated with this outcome are bed confinement, the use of drugs with sedative effects or neuromuscular blockers, and exposure to invasive mechanical ventilation (IMV) [1]. The clinical condition known as ICU-acquired weakness (ICU-AW) is defined as a syndrome that is not explained by any etiology other than ICU hospitalization and is associated with lowered quality of life [2].

The elderly population has a greater tendency to develop comorbidities, which contributes to hospital admissions. Polyneuropathy and myopathy related to intensive care are common conditions in these patients, generating a worse prognosis of ICU-AW [3, 4]. Respiratory muscles are among the most affected: muscle fibers can atrophy due to oxidative stress, activation of the ubiquitin-proteasome pathway, and a decrease in the number of myofibrils [5–7]. The muscles affected by IMV in the general population and especially in the elderly population include the diaphragm; when specific atrophy of the muscle fibers of the diaphragm is observed, the change is known as diaphragmatic dysfunction [8]. Controlling the factors that increase the risk of ICU-AW is key to preventing its development. Approximately 25-50% of subjects receiving ventilatory support have muscle weakness, and among this group, 85-95% experience neuromuscular impairment that persists for 2-5 years [9]. Considering the progression of diaphragmatic dysfunction, which is one manifestation of ICU-AW, consequences such as an increased IMV duration, an increased risk of respiratory complications, and a prolonged hospital stay may be observed if no specific intervention is performed [10, 11].

In the context of outpatient pulmonary rehabilitation, positive effects have been observed when using the transcutaneous electrical diaphragmatic stimulation (TEDS) technique in patients with chronic obstructive pulmonary disease (COPD) [12]. This technique consists of placing electrodes on the skin at locations near the motor points of the diaphragm, transmitting an intermittent current, and generating action potentials capable of producing muscle contractions [13, 14]. Studies evaluating the safety of neuromuscular electrical stimulation (NES) have suggested that it does not alter the stability of vital parameters such as heart rate (HR) and blood pressure (BP) [15, 16]. However, no study has evaluated the safety of TEDS in patients admitted to the ICU. Considering the practicality of the procedure, its low cost, the importance of applying new therapeutic modalities in the context of diaphragmatic dysfunction, and the scarcity of evidence on this topic in the ICU context, the objective of the present study was to evaluate the influence of TEDS on hemodynamic and vital parameters in critically ill elderly patients.

## 2. Materials and Methods

### 2.1. Study Design and Participants

This is an experimental study evaluating 47 patients (of 71 eligible) aged ≥60 years who underwent IMV at the Therezinha de Jesus Hospital and Maternity Hospital and at the Monte Sinai Hospital, both located in Juiz de Fora, Brazil. All participants underwent hemodynamic evaluations before and immediately after TEDS application. They underwent the same evaluation steps, and the variables systolic BP (SBP), diastolic BP (DBP), mean arterial pressure (MAP), and HR were measured immediately before and after the intervention. Patients with the following characteristics were excluded: a recent surgical scar and/or an open lesion in the regions where the electrodes would be placed, severe hemodynamic instability (HR > 140 bpm, MAP < 65 mmHg or>120 mmHg, peripheral oxygen saturation < 86% with a fraction of inspired oxygen ≥ 60%), patient–ventilator asynchrony not reversible with adjustments or optimization of sedation, hypoglycemia < 60 mg/dL, the presence of a cardiac pacemaker, untreated pneumothorax, and the use of increasing doses of vasoactive drugs.

This study was approved by the Research Ethics Committee of the Faculty of Medical and Health Sciences of Juiz de Fora (SUPREMA) under number 2.739.692, and all patients consented to participate in this study. This study was registered with the ClinicalTrials.gov identifier code NCT04565002.

### 2.2. Intervention

**Figure 1:**
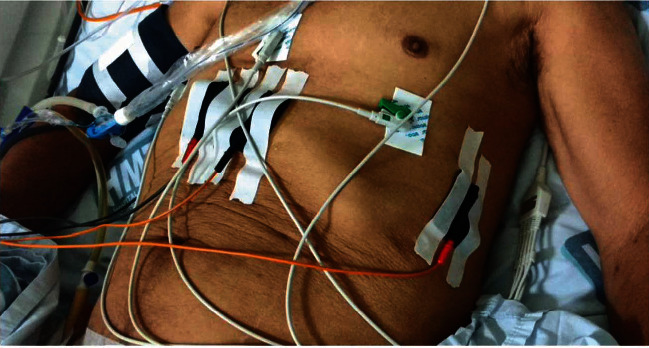
Positioning of the electrodes during transcutaneous electrical diaphragmatic stimulation.

The intervention was performed with the patient in assist-control ventilatory mode. The sensitivity parameter was adjusted to the value required for the contractions produced by the electrical stimulation not to trigger the mechanical ventilator, thus avoiding episodes of asynchrony. The following parameters were used: a frequency of 30 Hz, a pulse width of 0.4 ms, a respiratory rate (RR) of 15 breaths per minute, a hold time of 1 s, a rise time of 1 s, a fall time of 2 s, and a time without stimulation of 2 s [13]. Phrenic electrostimulation equipment (Dualpex 961, Quark, SP, Brazil) was used. The electrodes were positioned according to Cancelliero et al. [14], who proposed the placement of two electrodes in the right and left paraxiphoid regions and another two in the direction of the axillary midline on the seventh intercostal space on the right and left sides. The positioning of the TEDS electrodes is shown in Figure 1. Diaphragm stimulation lasted 30 minutes [12].

### 2.3. Data Analysis

The distribution of the measured outcome variables was evaluated by the Shapiro-Wilk test. The results are expressed as the mean (standard deviation (SD)) or frequency (percentage). The difference between the mean of a continuous variable before and after the intervention was evaluated by a paired *t*-test. The significance level adopted was *p* < 0.05. The data analysis was performed using the IBM SPSS Statistics version 23.0 software (IBM Corp., Armonk, NY, USA).

## 3. Results

Of the 71 patients who participated in the present study, 24 were excluded for the following reasons: interruption of a medical procedure (*n* = 5), intense psychomotor agitation (*n* = 12), and patient–ventilator asynchrony (*n* = 7). Thus, the sample consisted of 47 patients, including 33 males, with a mean age of 69.9 ± 7.64 years. The main clinical conditions leading to ICU admission were heart disease, lung disease, and sepsis. The characteristics of the sample, including demographic and anthropometric data and admission conditions, are shown in Table 1.

The mean SBP pre-TEDS was 126.6 ± 23.7 mmHg, whereas the value post-TEDS was 122.9 ± 25.9 mmHg (*p* = 0.467). The mean DBP values were 71.1 ± 12.2 mmHg before the procedure and 67.7 ± 14.2 mmHg after the intervention (*p* = 0.223). The comparisons between the pre-TEDS and post-TEDS values are shown in Table 2.

Power analysis was conducted using G∗power version 3.1. Considering a type-I error of 5%, a minimal correlation between measurements of 0.5, a two-tailed paired test, and the observed means ± SD (before and after TEDS), the actual statistical powers to detect the observed effects were 80% (SBP), 80.2% (DBP), 80% (MAP), and 80% (HR).

## 4. Discussion

This is the first study to evaluate the influence of TEDS on the hemodynamic and vital parameters of critically ill elderly patients. The main finding was no significant change in hemodynamic parameters in the sample from before to after TEDS application, suggesting that the intervention is safe.

NES is a safe technique when correctly applied by a trained professional [17–20]. When evaluating patients with COPD, Akar et al. [18] observed a reduction in HR in the postintervention period, suggesting that the use of NES did not generate cardiac overload in the patients evaluated. Iwatsu et al. [19] followed 61 patients in the postoperative period of cardiac surgery to study the safety of NES by evaluating hemodynamic parameters and the presence of arrhythmias. These authors did not observe changes in the evaluated parameters, suggesting that NES does not increase cardiac workload and is a safe intervention in these patients. In the present study, no statistically and clinically relevant difference in HR was noted when comparing pre- and post-TEDS time points. Even considering that the increase in transpulmonary pressure caused by IMV and muscle contractions affects cardiac output, right ventricular afterload, and left ventricular preload, TEDS did not change HR in our study, which can be at least partly explained by our adjustment of the sensitivity of the mechanical ventilator, thus preventing its triggering [21].

One of the concerns with the use of TEDS is the occurrence of adverse effects, including skin reactions. When evaluating critically ill patients, Segers et al. [20] did not observe a significant change in the studied variables (BP, HR, and RR), and only hyperemia occurred in 50% of the patients immediately after removal of the electrodes, which gradually disappeared. Our study did not find adverse events related to TEDS in the integumentary system, although the procedure lasted a short time. Additionally, regarding concerns about side effects, Parry et al. [22] used some parameters as safety cutoffs to determine when to start or stop NES. These authors did not observe serious adverse effects, corroborating the findings of the present study, which used some of their cutoff points.

Although few studies on TEDS have been published to date, this technique has been used in clinical practice in intensive physical therapy because it has shown promise for improving respiratory muscle strength [23–26]. Even with the physiological decline of the respiratory system resulting from aging, training with TEDS can be an effective tool in respiratory physiotherapy by promoting an increase in diaphragm muscle strength in elderly people [25]. The present study showed that TEDS did not significantly alter the hemodynamic parameters of this population, which reinforces its applicability in the ICU given the influence of the procedure on the motor units of the diaphragm that produce involuntary stimulation of the muscle and prevent autophagy and disuse atrophy. A recent systematic review [27] showed that the application of TEDS promotes an increase in respiratory muscle strength in individuals with COPD, elderly individuals, healthy women, and patients in the postoperative period of myocardial revascularization surgery.

Interestingly, a recent study conducted by Duarte et al. [28] suggested that TEDS influences the duration of IMV as well as the length of stay in the ICU in patients with spinal cord injury (SCI). Unlike the retrospective case series evaluated by these authors (*n* = 10), our study is an experimental clinical trial evaluating the influence of TEDS on immediate hemodynamic responses to determine the safety of the procedure in critically ill elderly individuals (not only patients with SCI). Additionally, Duarte et al. [28] proposed a comparison between TEDS and the standard weaning protocol in neurological patients who were already able to voluntarily contract their respiratory muscles. In contrast, we analyzed the application of TEDS in patients initially without ventilatory drive, thus allowing identification of new evidence to assess the impact of TEDS earlier, which is important because critical patients newly admitted to the ICU receive sedative and analgesic agents, justifying the decrease in the ventilatory drive and contributing to ICU-AW [29].

Immobility associated with IMV is one of the main factors involved in the development of ICU-AW and is a predictor of mortality, especially in the elderly population. Muscle strength can be lost at a rate of 10-15% per week of disuse. The combination of 18-69 hours of diaphragm inactivity and IMV is associated with marked atrophy of both slow- and fast-twitch fibers [30]. Kress and Hall [30] suggested that increased proteolysis, which is responsible for fiber atrophy, can be attenuated and thus improve the outcomes of this specific population. These data demonstrate the importance of measures to prevent the consequences of inactivity, such as the intervention proposed in our study. We believe that ICU-AW-preventive measures in elderly patients, such as TEDS, can become part of the routine of ICUs to forestall unfavorable outcomes in this population.

The main limitations were the fact that the procedure was applied only once, the lack of a control group, and the lack of assessment of the effect of the technique on the diaphragm muscle strength. Although no significant difference was found in the variables studied before and after the procedure, the small sample size and the different clinical conditions of hospitalization should be highlighted; these are limiting factors for predicting the safety of TEDS in critically ill elderly patients. In addition, given our use of a single evaluation step, the medium- or long-term effects of the intervention could not be identified. Despite these limitations, we believe that this study can serve as a starting point for controlled and randomized clinical trials that can deepen our knowledge about TEDS.

## 5. Conclusions

TEDS did not have clinically relevant impacts on hemodynamic or vital parameters when comparing the pre- and postintervention time points in critically ill elderly patients. These findings point to the possible safety of TEDS application in this population.

## Figures and Tables

**Table 1:** Characteristics of the studied sample.

Variables	Values
Demographic data	
Age (years)	69.9 ± 7.64
Sex (male)	33 (70.2%)
Body composition	
Weight (kg)	64.6 ± 9.87
Height (cm)	166.5 ± 6.85
Clinical conditions requiring hospitalization	
Cardiac diseases	
Acute heart failure	6 (12.8%)
Acute myocardial infarction	4 (8.51%)
Lung diseases	
COPD exacerbation	5 (10.6%)
Asthma exacerbation	5 (10.6%)
Neurological diseases	
Stroke	3 (6.38%)
Spinal cord injury	3 (6.38%)
Sepsis	9 (19.2%)
Postoperative state after cardiac surgery	7 (14.9%)
Postoperative state after abdominal surgery	5 (10.6%)

Results expressed as the means ± SD or number (%).

**Table 2:** Differences between systolic blood pressure, diastolic blood pressure, mean arterial pressure, and heart rate from before to after the intervention.

Variables	Before TEDS	After TEDS	*p* value
SBP	126.6 ± 23.7	122.9 ± 25.9	0.467
DBP	71.1 ± 12.2	67.7 ± 14.2	0.223
MAP	90.2 ± 16.7	86.7 ± 18.8	0.335
HR	92.8 ± 18.9	93.5 ± 19.2	0.846

Results expressed as the means ± SD. SBP: systolic blood pressure; DBP: diastolic blood pressure; MAP: mean arterial pressure; HR: heart rate; TEDS: transcutaneous electrical diaphragmatic stimulation.

## Data Availability

The data used to support the findings of this study are available from the corresponding author upon reasonable request.
